# Fulminant Methicillin-Sensitive Staphylococcus aureus Pneumonia in a Steroid-Treated Patient With End-Stage Renal Disease: A Rapidly Fatal Case

**DOI:** 10.7759/cureus.84155

**Published:** 2025-05-15

**Authors:** Satoshi Yoshizaki, Keiki Shimizu

**Affiliations:** 1 Department of Emergency and Critical Care Medicine, Tokyo Metropolitan Tama Medical Center, Tokyo, JPN; 2 Department of Emergency and Critical Care Medicine, ECMO Center, Tokyo Metropolitan Tama Medical Center, Tokyo, JPN

**Keywords:** antibiotic administration in management of severe sepsis, cardiac pulmonary arrest (cpa), chronic methylprednisolone therapy, continuous hemodialysis and filtration, fulminant methicillin-sensitive staphylococcus aureus pneumonia, gouty nephropathy, hemodialysis, organ failure from sepsis, panton–valentine leukocidin (pvl)-associated s. aureus infection, venoarterial extracorporeal membrane oxygenation (va-ecmo)

## Abstract

A man in his 50s with end-stage renal disease (ESRD) secondary to gouty nephropathy and on chronic methylprednisolone therapy presented with acute-onset weakness, severe hyperkalemia, metabolic acidosis, and lactic acidemia. Emergency hemodialysis was initiated; however, within hours, he developed respiratory failure and progressive shock. Imaging revealed rapidly evolving right-dominant pneumonia. Despite escalation to broad-spectrum antibiotics, mechanical ventilation, and veno-arterial extracorporeal membrane oxygenation (VA-ECMO), the patient died within 28 hours of admission.

An autopsy revealed fulminant necrotizing pneumonia due to methicillin-sensitive *Staphylococcus aureus* (MSSA), with Gram-positive cocci present in the bronchial lumen and necrotic tissue. Histological findings included bronchial wall destruction, pulmonary edema, and alveolar hemorrhage. Additional findings included bilateral renal atrophy with arteriosclerosis, but no evidence of gouty tophus or urate deposition.

This case illustrates the potential for MSSA to cause rapidly progressive necrotizing pneumonia in immunocompromised hosts. The fulminant nature of the disease emphasizes the importance of early recognition, consideration of toxin-producing strains such as PVL-positive *S. aureus*, and the initiation of appropriate antimicrobial and supportive therapy. Despite aggressive interventions, the patient succumbed to multiorgan failure, highlighting the lethality of this condition in vulnerable populations.

## Introduction

Patients with end-stage renal disease (ESRD) are at increased risk of severe infections, especially when immunosuppressed [1,2]. Methicillin-sensitive *Staphylococcus aureus* (MSSA) pneumonia, though less frequently reported than methicillin-resistant *S. aureus* (MRSA) pneumonia, is recognized in specific high-risk populations such as the elderly, immunocompromised individuals, and those with chronic illnesses, including diabetes and renal failure [3]. It can also occur as a secondary bacterial infection following viral illnesses such as influenza. We present a case of rapidly fatal MSSA pneumonia in a patient with ESRD on long-term corticosteroid therapy. The case underscores the diagnostic challenges, aggressive disease course, and importance of early intervention in immunocompromised hosts.

## Case presentation

A man in his 50s with a history of gout, hypertension, chronic kidney disease, and peptic ulcer was being followed at our nephrology outpatient clinic. He had been diagnosed with ESRD secondary to gouty nephropathy and was scheduled to initiate maintenance dialysis the following day (day x + 1). Past medical history included hyperuricemia, hypertension, chronic kidney disease, and gastric ulcer. There was no known drug allergy. Regular medications included oral methylprednisolone 8 mg daily, acetaminophen granules 3000 mg, colchicine 1 mg, daprodustat 12 mg, sodium zirconium cyclosilicate hydrate 30 g (powder form), alfacalcidol 1 µg, and ferric citrate hydrate 750 mg. He had been taking oral methylprednisolone 8 mg daily for chronic inflammation, specifically for multiple tophi due to gout, and had been on corticosteroid therapy for at least two years, placing him at increased risk for infection due to immunosuppression [1,2].

**Figure 1:**
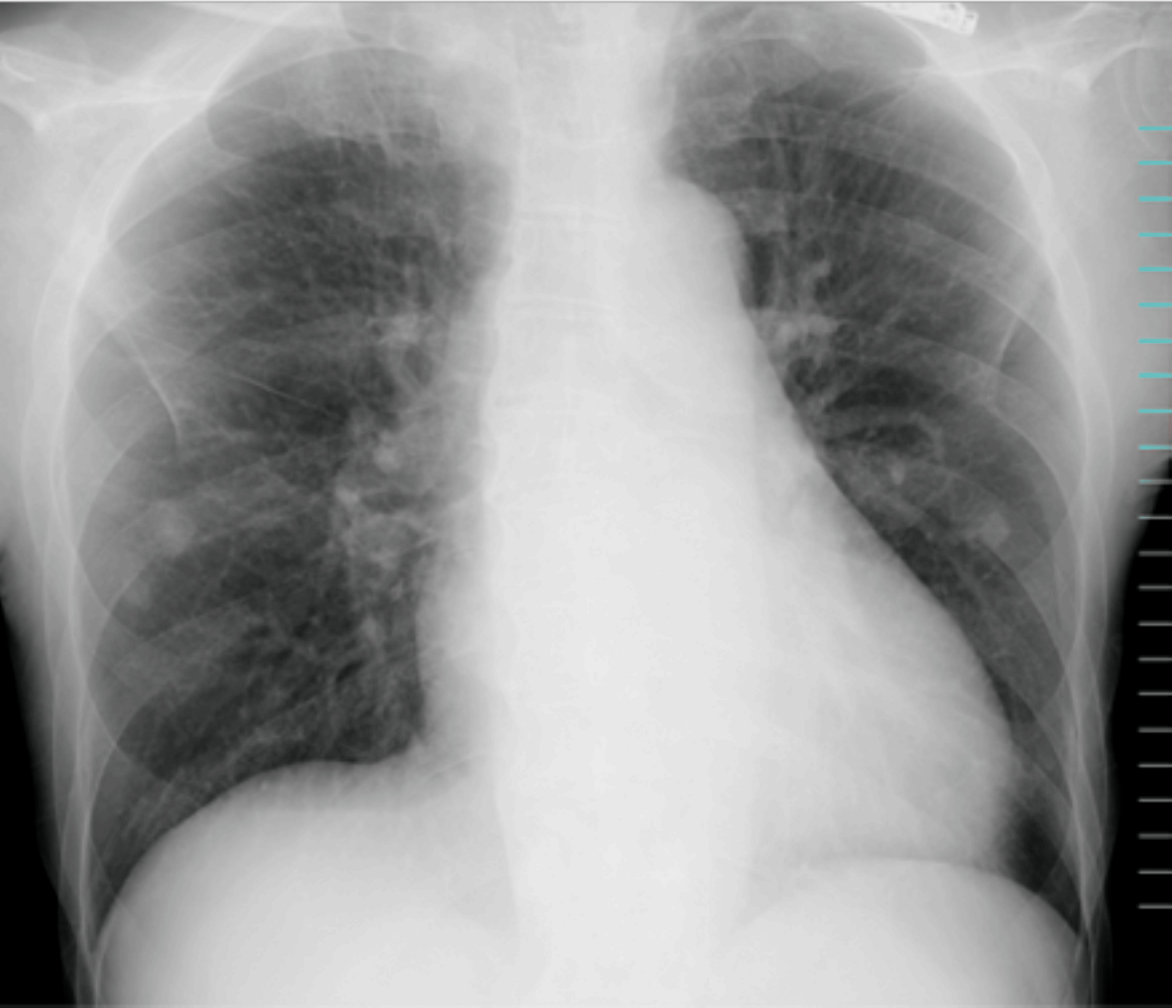
Initial chest X-ray showing clear lung fields and sharp costophrenic angles

**Figure 2:**
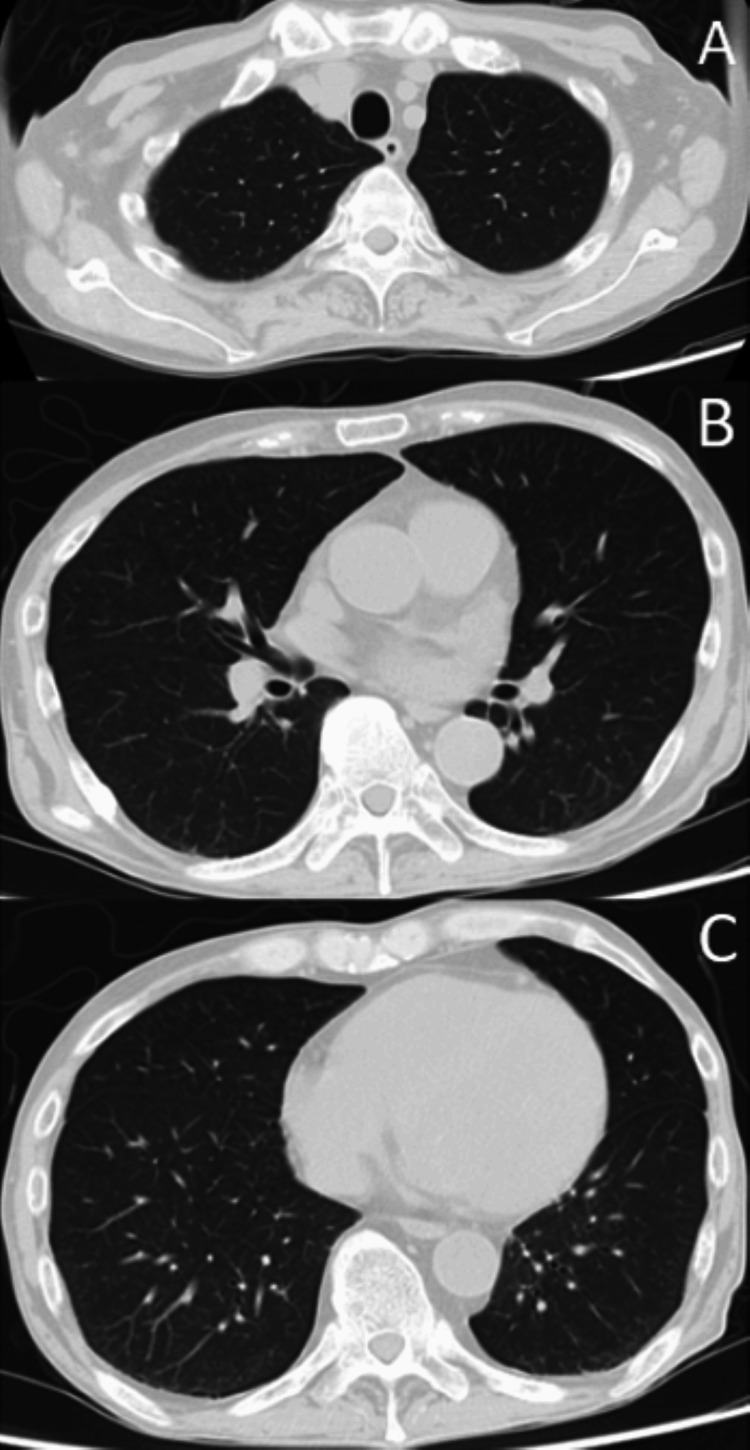
Axial chest CT image at (A) the upper lung fields, (B) the mid-thorax, and (C) the lower lung fields The chest CT scan at admission shows no signs of consolidation or pleural effusion.

**Figure 3:**
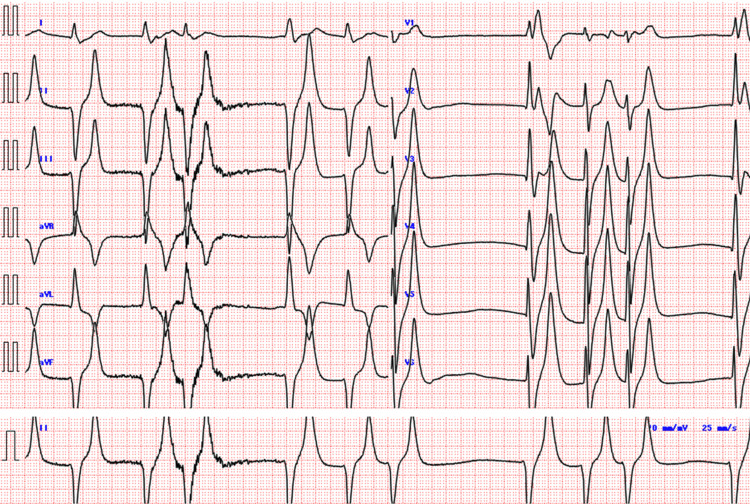
ECG showing bradycardia, wide QRS complexes, and peaked T waves, findings consistent with hyperkalemia

On day x at 5:00 a.m., he presented to the emergency department (ED) with a sudden onset of profound weakness and difficulty moving after experiencing limb weakness and falling at home. His vital signs on arrival were as follows: GCS, E4V5M6; temperature, 36.3°C; respiratory rate, 16/min; heart rate, 95 bpm; blood pressure, 197/161 mmHg; and SpO₂, 91% on room air. He exhibited no peripheral edema or abnormal pulmonary sounds. A chest X-ray showed clear lung fields with sharp costophrenic angles (Figure 1), and a CT scan showed no pulmonary consolidation or pleural effusion (Figure 2). Echocardiography revealed diffuse hypokinesia with a visually estimated ejection fraction in the 30% range. ECG demonstrated bradycardia with absent P waves, wide QRS complexes, a ventricular escape rhythm, and peaked T waves suggestive of hyperkalemia (Figure 3). Despite laboratory findings suggestive of significant inflammation and possible infection (Tables 1, 2), we initially anticipated that hemodialysis would effectively correct the hyperkalemia, allowing for the continuation of maintenance dialysis.

**Table 1:** Laboratory results overview

Parameter	Value	Unit
Complete blood count (CBC)		
WBC	14,700	/μL
RBC	4.37 × 10^6^	/μL
HGB	12.2	g/dL
HCT	41.9	%
MCV	95.9	fL
MCH	27.9	pg
MCHC	29.1	g/dL
PLT	189,000	/μL
Coagulation profile		
PT-INR	1.22	–
APTT	32.2	sec
D-dimer	22.1	μg/mL
Biochemistry		
TP	6.5	g/dL
Albumin	3.5	g/dL
BUN	215.6	mg/dL
Creatinine	9.98	mg/dL
eGFR	5	mL/min/1.73 m^2^
NH₃	159	μg/dL
Na	130	mmol/L
Cl	100	mmol/L
K	9.1	mmol/L
P	13.4	mg/dL
AST	85	U/L
ALT	23	U/L
LDH	1133	U/L
ALP	184	U/L
Glucose	69	mg/dL
CRP	23.45	mg/dL
Venous blood gas analysis (VBG)		
Hb	12.3	g/dL
pH	6.846	–
PO₂	39.5	mmHg
PCO₂	30.8	mmHg
Base excess	-28.5	mmol/L
HCO₃⁻	5	mmol/L
Na^+^	127	mmol/L
K^+^	8.5	mmol/L
Ca²^+^	0.86	mmol/L
Cl⁻	105	mmol/L
Anion gap	16.1	mmol/L
Glucose	69	mg/dL
Lactate	4.4	mmol/L

**Table 2:** The viral panel tests were all negative HIV antigen/antibody, SARS-CoV-2 antigen quantitative test, multiplex PCR, and *Legionella*/*Streptococcus pneumoniae* antigen were all negative.

Test	Result
HIV Ag + Ab (index)	0.7
HIV Ag + Ab (result)	(-)
SARS-CoV-2 antigen result	(-)
SARS-CoV-2 antigen concentration	<0.60
SARS-CoV-2 PCR	(-)
Adenovirus	(-)
Coronavirus 229E	(-)
Coronavirus HKU1	(-)
Coronavirus NL63	(-)
Coronavirus OC43	(-)
Human metapneumovirus	(-)
Rhinovirus/enterovirus	(-)
Influenza A	(-)
Influenza B	(-)
Parainfluenza 1	(-)
Parainfluenza 2	(-)
Parainfluenza 3	(-)
Parainfluenza 4	(-)
RS virus	(-)
Bordetella pertussis	(-)
Chlamydia pneumoniae	(-)
Mycoplasma pneumoniae	(-)
*Legionella* antigen (urine)	(-)
*Streptococcus pneumoniae* antigen (urine)	(-)

Immediate management included oxygen administration (3 L/min via reservoir mask), intravenous calcium gluconate, and insulin-glucose therapy (40 mL of 50% dextrose with four units of regular insulin), which resolved the ventricular tachycardia. Due to elevated inflammatory markers and suspicion of infection, contrast-enhanced chest CT was performed, which showed no obvious pulmonary pathology at that time. He was admitted to the high care unit (HCU) for emergent hemodialysis. Severe hyperkalemia and metabolic acidosis are well-established indications for urgent renal replacement therapy [4,5].

Hemodialysis was initiated promptly. However, fluid overload was not apparent, and ultrafiltration was limited to 1160 mL due to hypotension. Post-dialysis labs showed persistent lactic acidosis (lactate, 9.9 mmol/L), prompting continuation of care in the emergency intensive care setting.

On day x + 1 at 1:00 a.m., he reported dyspnea, with a respiratory rate of 30/min and SpO₂ 86% on 3 L/min oxygen. Oxygen flow was increased to 5 L/min, resulting in transient improvement (SpO₂ 99%). However, at 5:30 a.m., he again experienced respiratory distress, with SpO₂ decreasing to 90% on 5 L/min. Repeat blood gas analysis showed further lactate elevation (11.3 mmol/L), persistent hyperkalemia, and hypoglycemia. These were managed with dextrose and bicarbonate infusion.

**Figure 4:**
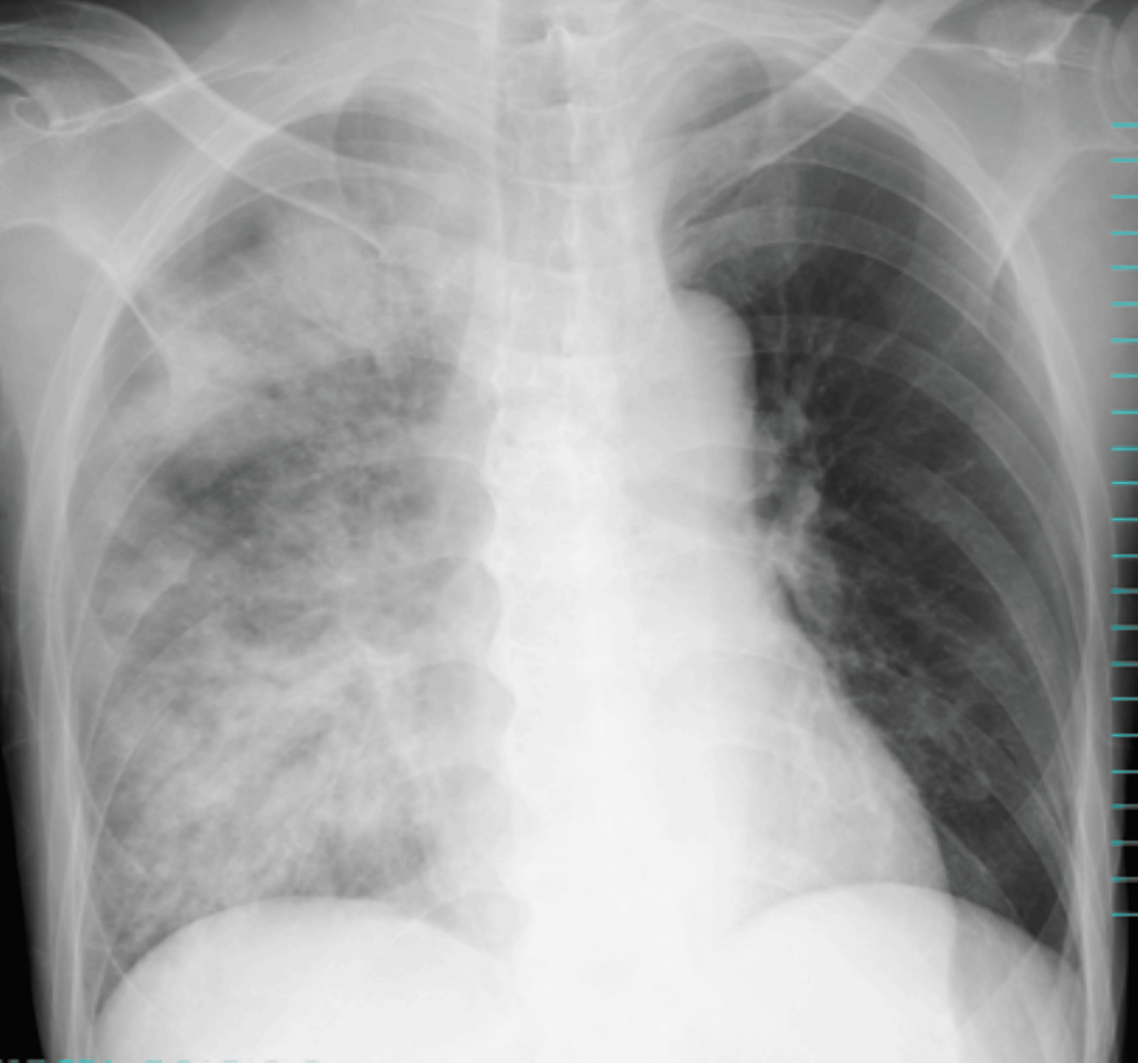
Chest X-ray on day x + 1 at 6:12 a.m. showing diffuse right lung opacity

At 6:12 a.m., despite 5 L/min of oxygen, his SpO₂ remained at 85%. A repeat chest X-ray revealed marked decreased transparency in the entire right lung field (Figure 4), raising suspicion of pneumonia. Oxygen therapy was escalated to 10 L/min, and ceftriaxone (2 g/day) was initiated.

**Figure 5:**
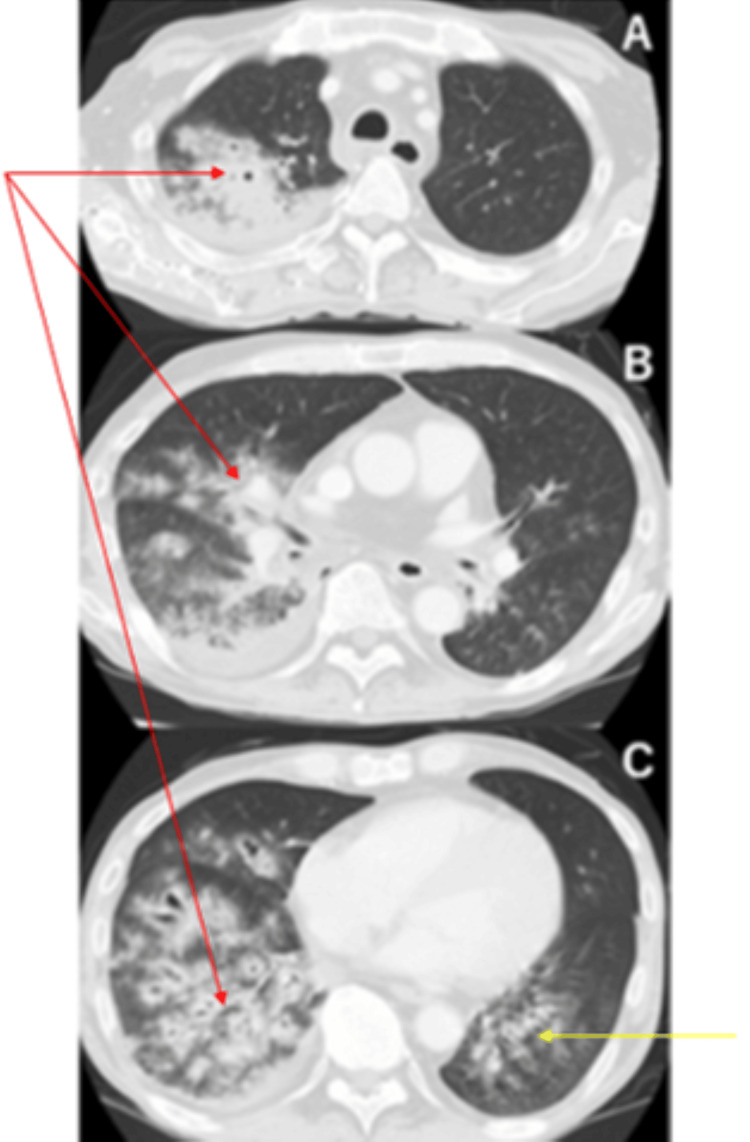
Follow-up axial CT on day x + 1 at (A) the upper lung fields, (B) the mid-thorax, and (C) the lower lung fields Follow-up axial CT on day x + 1 shows bilateral bronchocentric consolidation and ground-glass opacities. New bronchial dilation is visible, especially in the right lung (red arrow), consistent with necrotizing bronchopneumonia. A yellow arrow indicates a newly formed cavity within the area of consolidation, further supporting the diagnosis of necrosis.

A follow-up chest X-ray taken at 9:28 a.m. revealed progression of right lung opacities. On the left lower lung field, overlapping the cardiac silhouette, there was inhomogeneous decreased translucency with visible air bronchograms. A concurrent chest CT (Figure 5) scan demonstrated widespread consolidation and ground-glass opacities along the bronchovascular bundles in the dorsal segment of the right upper lobe, the right middle and lower lobes (red arrow), and the dorsal segment of the left lower lobe (yellow arrow). The lesions appeared to spread centrifugally from the bronchi, suggestive of bronchogenic dissemination. Compared with imaging from day 1, newly emerging bronchial dilatation was noted, particularly in the intermediate bronchi of the right upper lobe, segment 5 of the right middle lobe, and the lower lobe bronchi. The speed of progression was too rapid to be explained by traction bronchiectasis alone, raising the possibility of bronchial wall destruction due to a highly virulent pathogen. Interlobular septal thickening was noted in parts of the right lung and the left lower lobe.

At 10:30 a.m., he was intubated for worsening hypoxemia (SpO₂ 85% on 10 L/min O₂). At 12:41 p.m., he suffered cardiac arrest with pulseless electrical activity (PEA); return of spontaneous circulation (ROSC) was transiently achieved before re-arrest occurred. Veno-arterial extracorporeal membrane oxygenation (VA-ECMO) was initiated [6,7,8].

**Figure 6:**
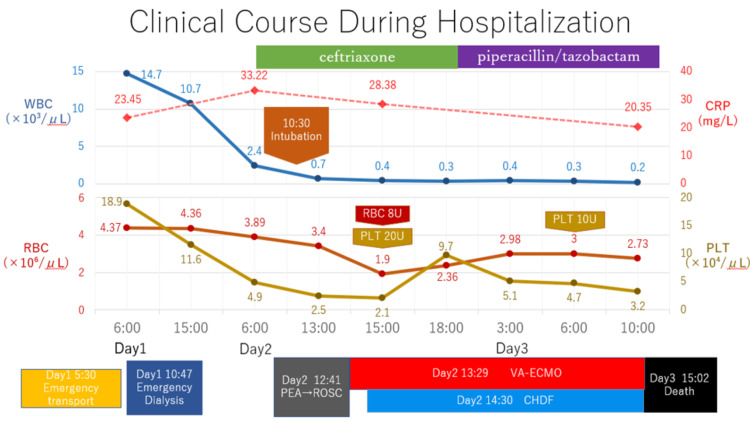
Clinical course from admission to death showing WBC, CRP, SpO₂, transfusion, antibiotics, mechanical support, and general condition

**Figure 7:**
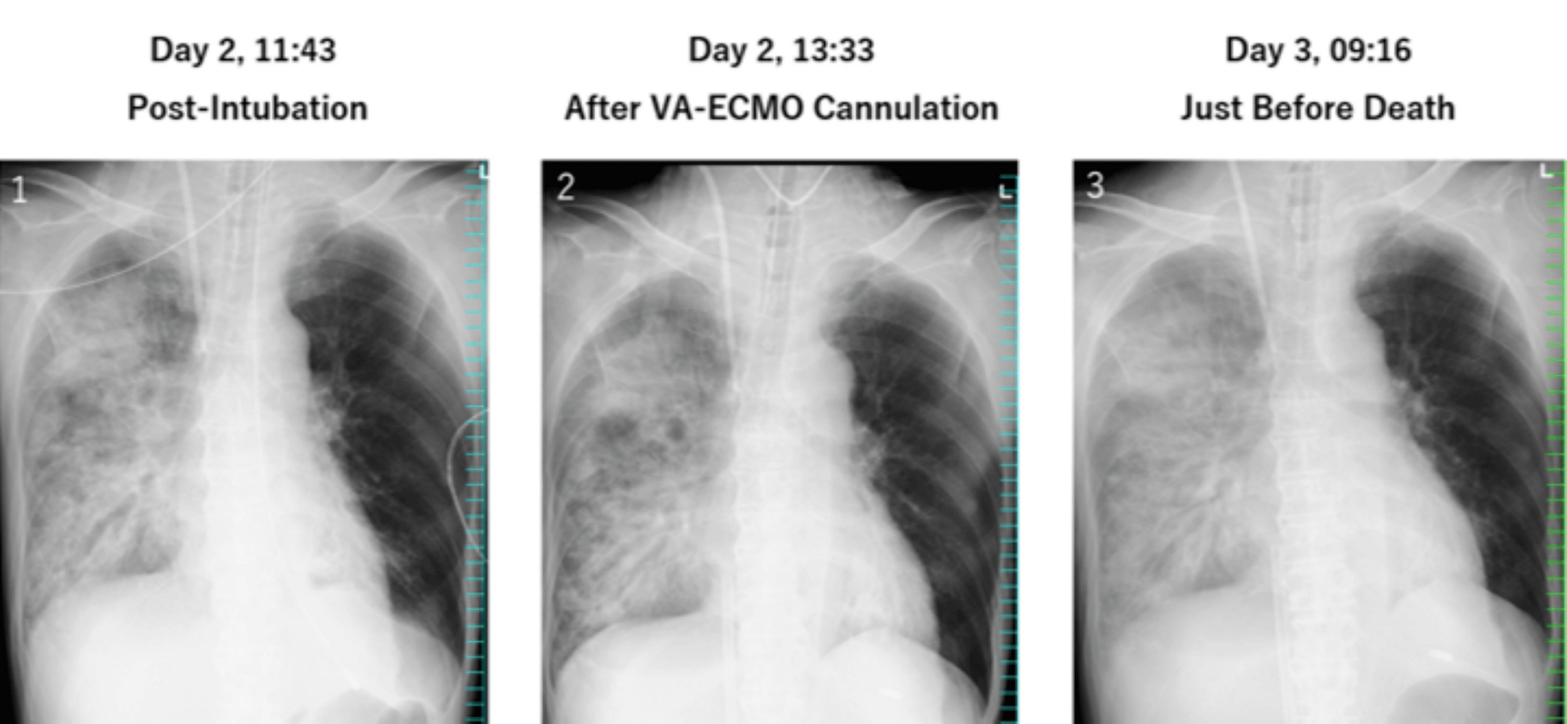
Sequential chest X-ray changes and rapid progression of unilateral pulmonary consolidation (Slide 1) Day 2 at 11:43 a.m., post-intubation: chest X-ray after endotracheal intubation. Initial unilateral pulmonary consolidation was noted. (Slide 2) Day 2 at 13:33 p.m., after VA-ECMO cannulation: chest X-ray after veno-arterial extracorporeal membrane oxygenation (VA-ECMO) initiation. Increasing bronchial wall thickening was noted. (Slide 3) Day 3 at 09:16 a.m., just before death: final chest X-ray taken shortly before death. Marked bronchial dilatation consistent with necrotizing pneumonia.

Continuous hemodiafiltration (CHDF) was started due to refractory hyperkalemia. Despite transfusion of red blood cells and platelets and broadening of antimicrobial therapy, the patient developed pancytopenia and progressive multi-organ failure. Peripheral mottling was observed, and increasing doses of noradrenaline (up to 0.3 μg/kg/min) were required. Despite consideration of veno-arteriovenous ECMO (VAV-ECMO), his condition continued to deteriorate. With the family present, ECMO support was withdrawn, and death was confirmed at 15:02 p.m. on day x + 1, approximately 28 hours after admission (Figures 6, 7) [9].

Microbiological and biomarker results and postmortem findings

Blood cultures (two sets) demonstrated no growth, whereas the sputum culture was positive for MSSA. Notably, the patient tested positive for Mycoplasma pneumoniae IgM, which initially raised the possibility of atypical pneumonia. However, all other serological and biomarker tests, including cytomegalovirus antigenemia, β-D-glucan, Cryptococcus antigen, and Aspergillus antigen, were negative, as were autoantibodies. In conjunction with the postmortem histopathological examination, these microbiological findings confirmed that necrotizing pneumonia caused by MSSA was the definitive cause of death.

Postmortem findings

A complete autopsy was performed two days and 23 hours after death.

Macroscopic findings

**Figure 8:**
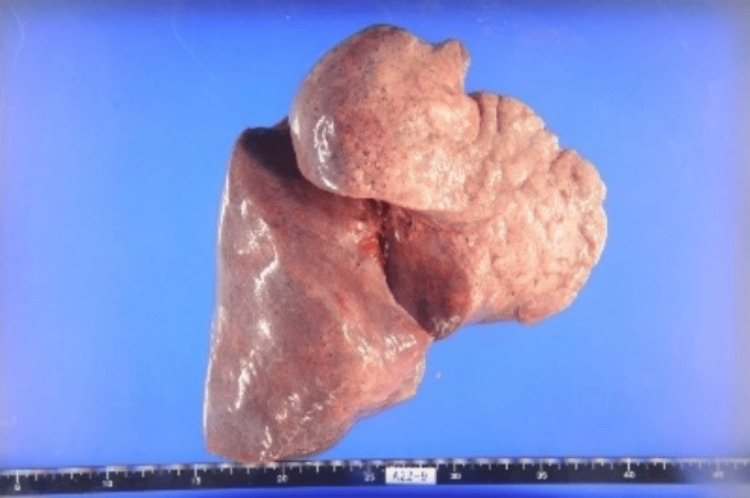
Gross appearance of the right lung showing increased weight and pale reddish coloration consistent with congestion

The right lung was markedly congested and edematous, weighing well above the expected range. It appeared pale reddish, suggesting pulmonary congestion. A large volume of serous (lightly blood-tinged) pleural effusion was present (average 559 g) (Figure 8). The left lung also demonstrated increased weight and similar serous effusion (average 492 g).

**Figure 9:**
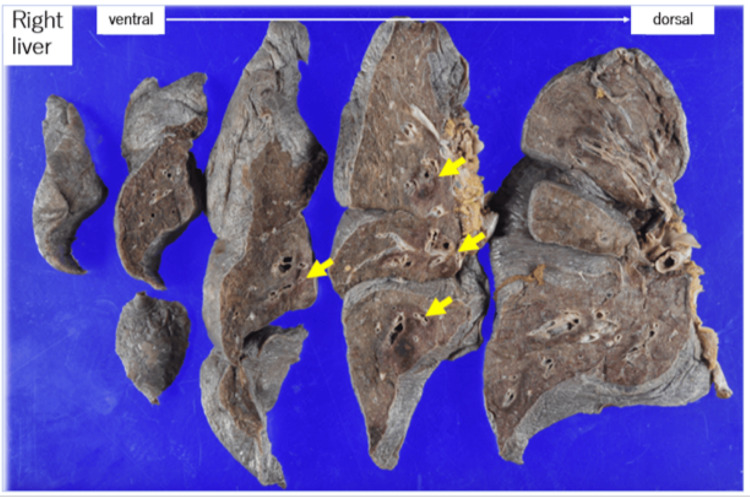
Formalin-fixed cut surface of the right lung showing peribronchial hemorrhage (yellow arrow)

On the formalin-fixed cut surface of the right lung, hemorrhagic changes were evident, particularly surrounding the bronchial tree (Figure 9). The left lung showed similar peribronchial hemorrhages.

Microscopic findings

**Figure 10:**
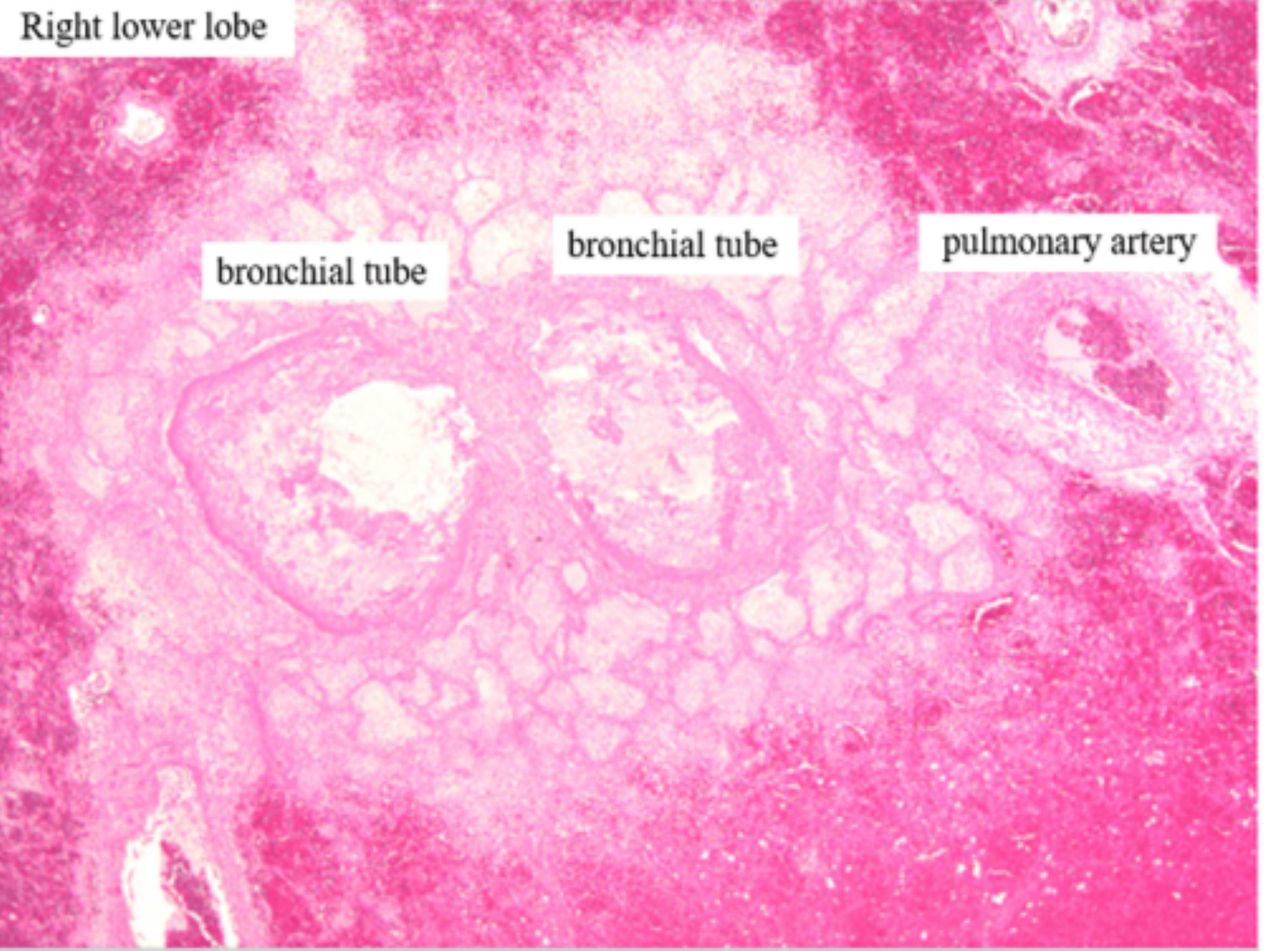
Histology of the right lower lobe showing alveolar hemorrhage and intra-bronchial bacterial aggregates in the airway (H&E stain)

Histological examination of the right lower lobe demonstrated marked pulmonary edema and alveolar hemorrhage, with clusters of bacterial colonies visible in the bronchial lumen (Figure 10). This is consistent with bronchopneumonia, often seen in fulminant *S. aureus* infections [1].

**Figure 11:**
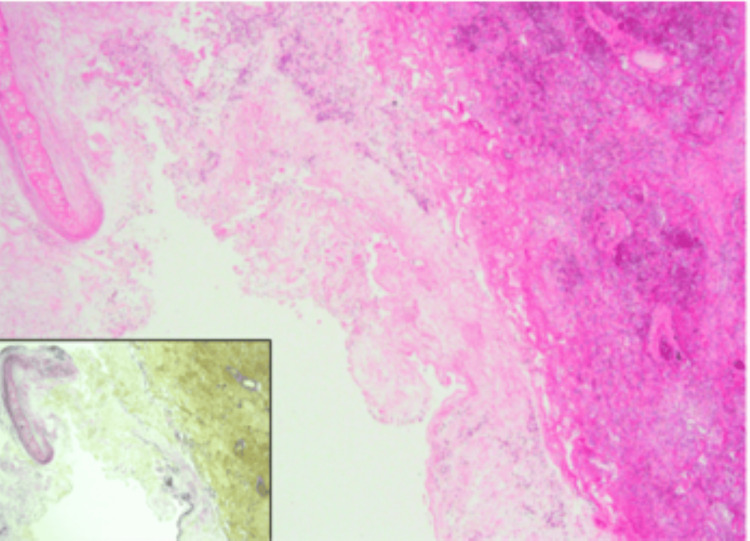
Prominent pulmonary edema and hemorrhage around the bronchus, intraluminal bacterial colonies suggesting bronchopneumonia A bacterial aggregate-like structure is visible within the bronchial lumen. These findings support the diagnosis of bronchopneumonia associated with acute airway infection.

In the bronchiectatic region of the right lower lobe, the bronchial wall exhibited edematous thickening and necrosis (Figure 11).

**Figure 12:**
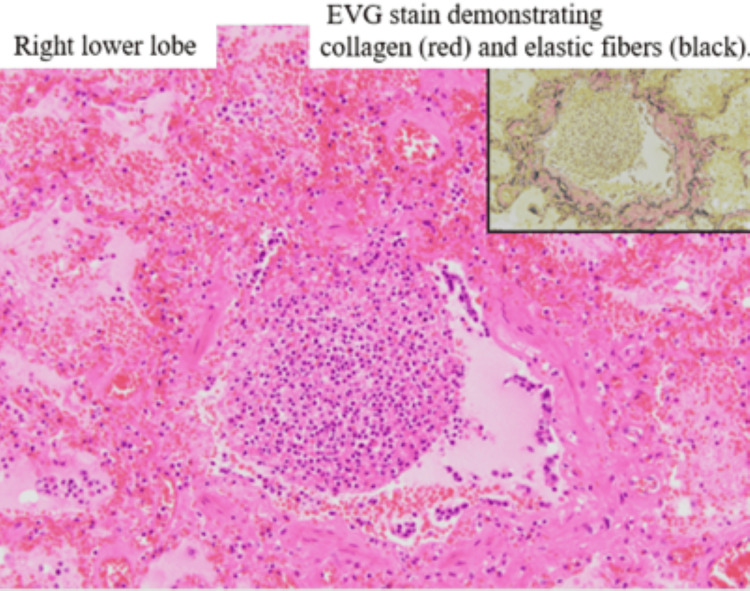
High-power EVG stain of the bronchus This is a high-power view of the peribronchial region in the right lower lobe. The inset shows Elastic van Gieson (EVG) staining, where collagen fibers appear red and elastic fibers appear black. Neutrophilic infiltration is observed within the bronchiole, and neutrophils are also present within the surrounding alveolar spaces.

Elastic Van Gieson (EVG) staining showed significant disorganization of elastic fibers, indicating architectural destruction of the bronchial wall, a feature commonly seen in necrotizing pneumonia [2]. High-power images showed dense neutrophilic infiltration in the bronchioles and adjacent alveoli (Figure 12).

**Figure 13:**
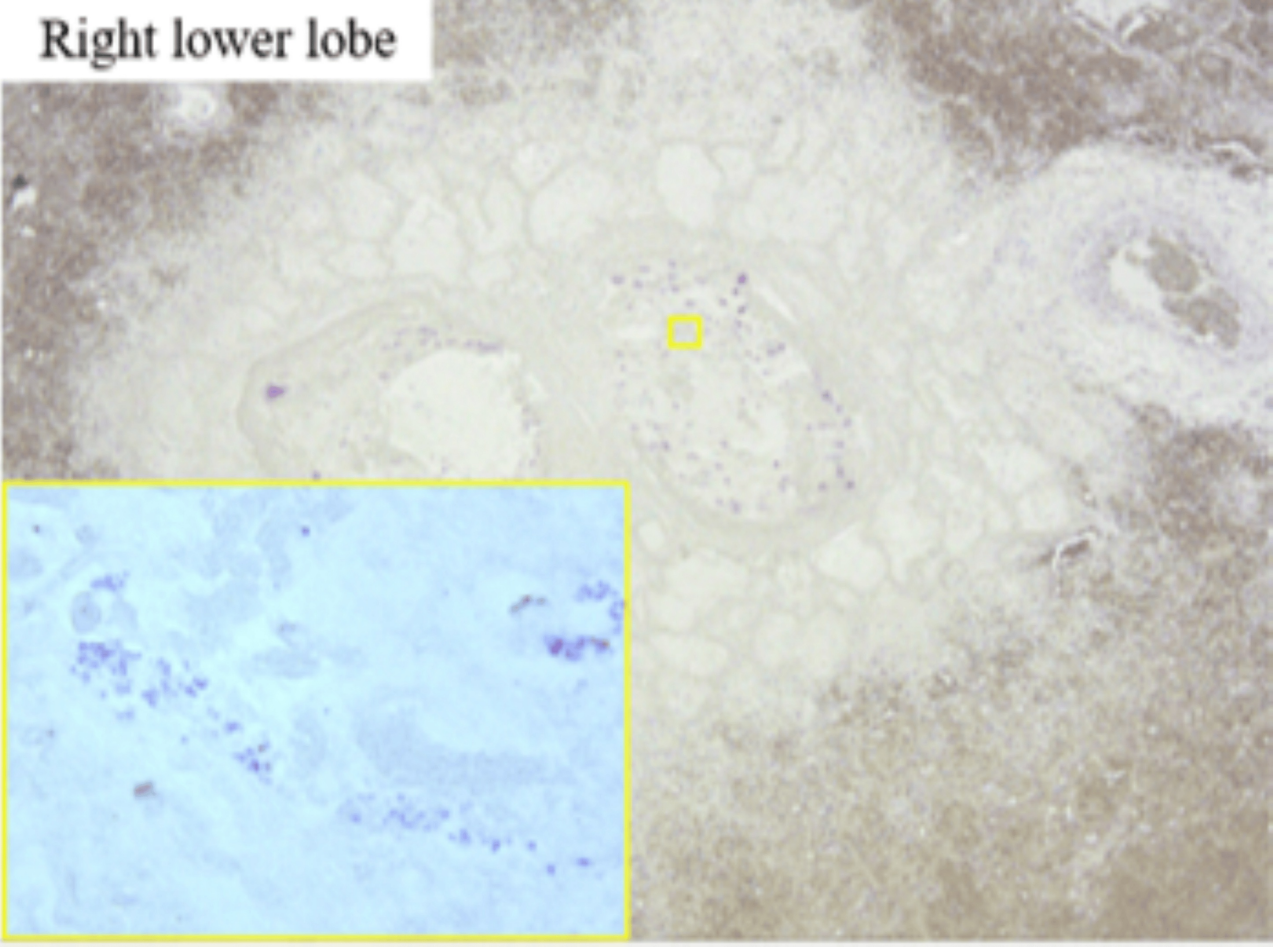
Gram stain showing Gram-positive cocci in the bronchial lumen Gram stain of the bronchial lumen in the right lower lobe. The inset shows a high-power magnification of the bronchial space, revealing numerous Gram-positive cocci within the airway. These findings are consistent with bronchopneumonia. Similar findings were observed in the left lung.

Gram staining confirmed the presence of gram-positive cocci within the bronchial lumen (Figure 13), supporting the diagnosis of severe *S. aureus*-associated bronchopneumonia [1,3]. These findings are consistent with bronchopneumonia. Similar findings were observed in the left lung.

Renal findings

Gross examination of both kidneys showed bilateral atrophy. A 1-cm solid nodule was identified at the lower pole of the right kidney. Histology of this mass revealed a solid proliferation of mildly atypical epithelial cells with clear cytoplasm, including areas of papillary architecture. Despite postmortem degradation, immunohistochemical staining was PAX8 (±), CD10 (+), and Vimentin (+), supporting the diagnosis of clear cell renal cell carcinoma.

**Figure 14:**
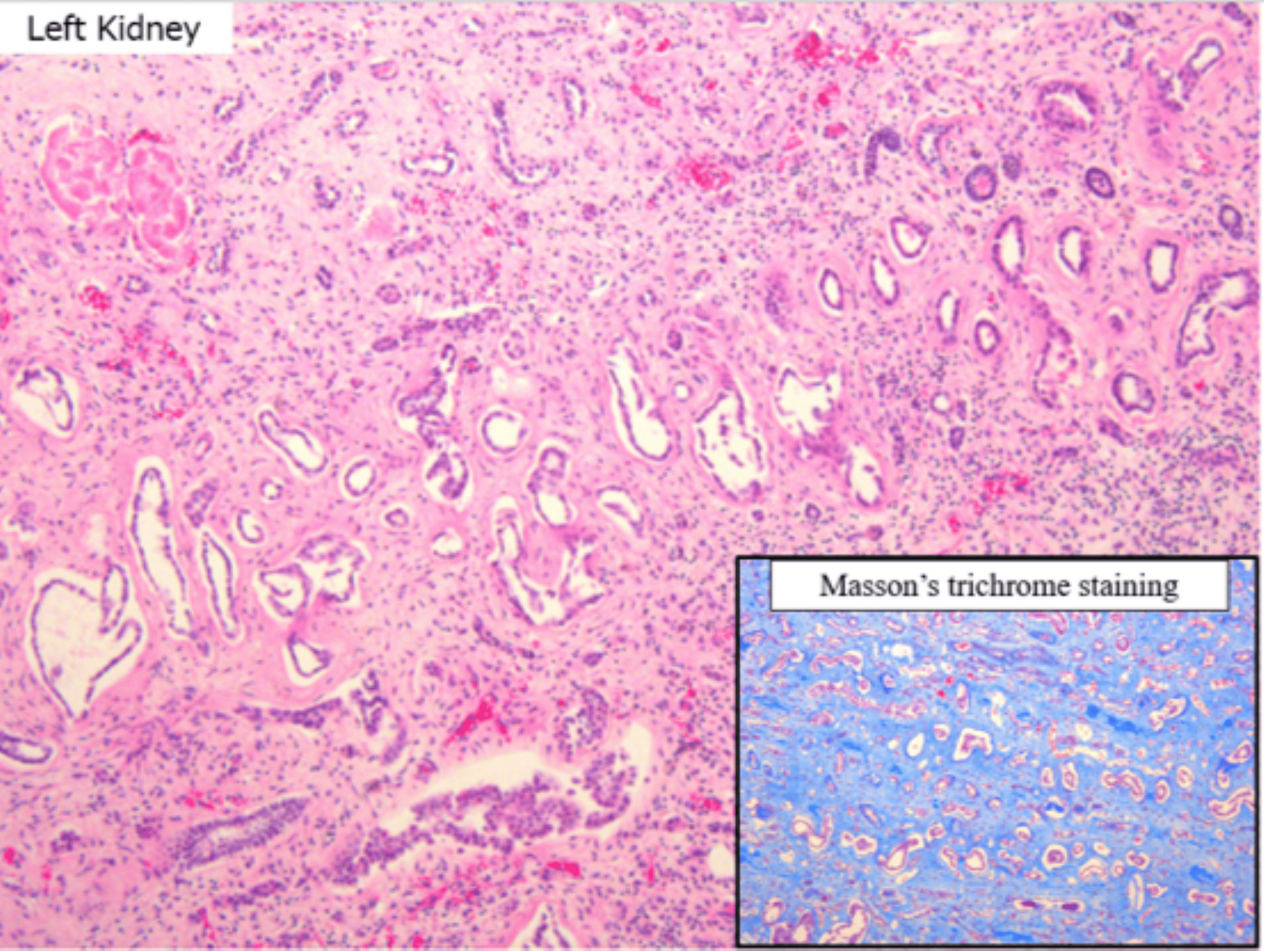
Renal histology shows tubular atrophy and interstitial fibrosis (blue in Masson’s trichrome), with moderate lymphocyte infiltration Histopathological findings of the kidney. The tubules show marked atrophy, and the interstitium exhibits severe fibrosis. The inset displays Masson’s trichrome staining, with fibrotic areas stained blue. Moderate lymphocytic infiltration is also noted within the interstitium.

The left kidney revealed extensive glomerulosclerosis, marked tubular atrophy, and interstitial fibrosis (Figure 14). Masson’s trichrome staining confirmed widespread fibrosis, and moderate lymphocytic infiltration was noted.

**Figure 15:**
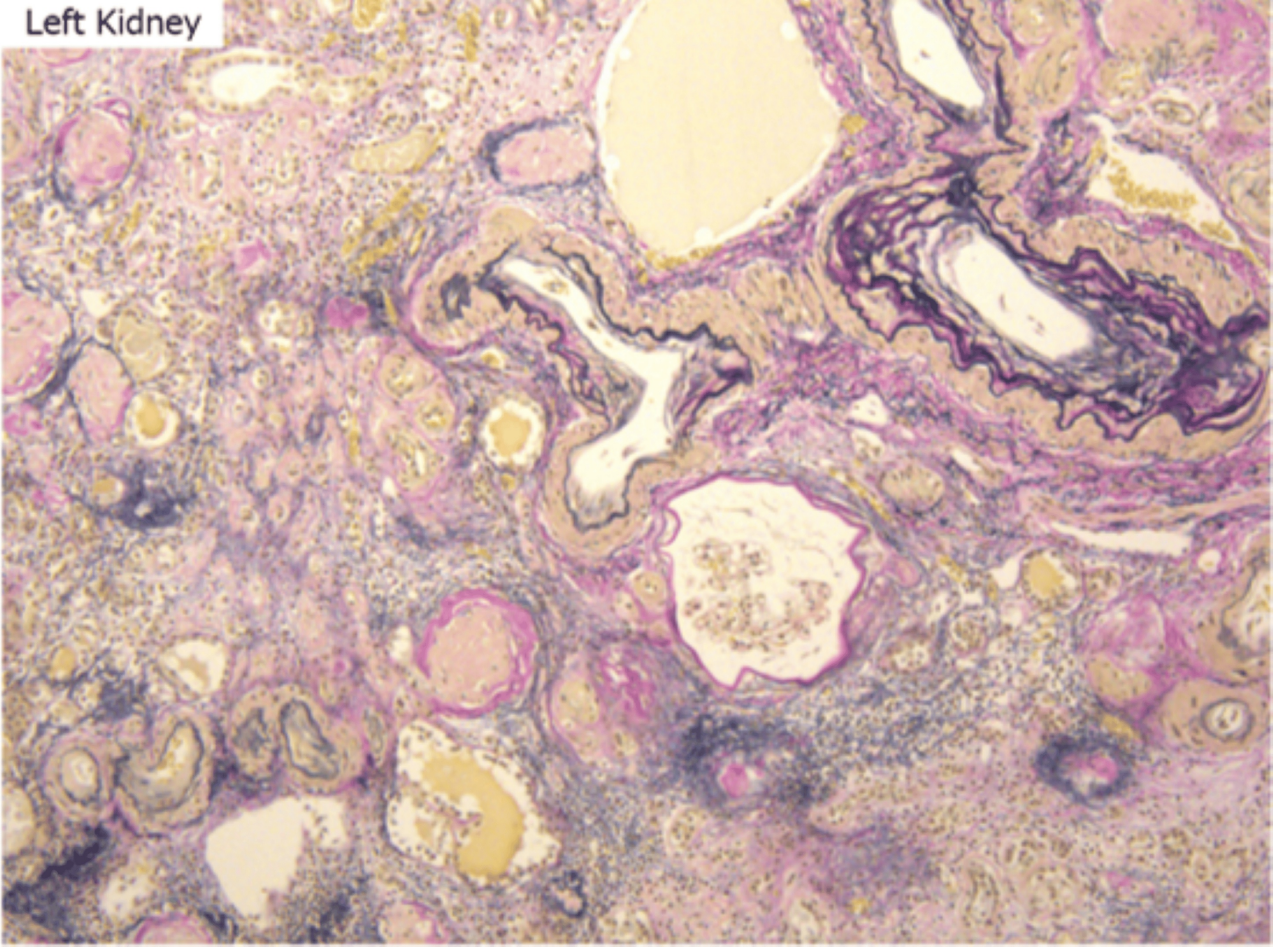
Arcuate/interlobular arteries with multilayered internal elastic lamina and narrowed lumens, indicating arteriosclerosis Histological image showing multilayering of the internal elastic lamina and marked luminal narrowing in arcuate to interlobular arteries, consistent with arteriosclerosis. Similar findings were observed in both kidneys. These vascular changes support a diagnosis of hypertensive nephrosclerosis and chronic kidney disease due to arteriosclerosis. No definitive tophaceous lesions suggestive of gout were identified.

Arteriosclerosis was prominent in arcuate and interlobular arteries, with duplication of the internal elastic lamina and luminal narrowing (Figure 15). These findings are consistent with hypertensive nephrosclerosis and chronic kidney disease. No evidence of urate crystal deposition or tophaceous gout was observed.

Autopsy diagnosis

The immediate cause of death was respiratory failure. The primary pathological findings included bronchopneumonia (with the right lung more severely affected than the left), pulmonary edema, alveolar hemorrhage, and clear cell renal cell carcinoma. Secondary findings comprised serous pleural effusions (approximately 300 mL on the left and 600 mL on the right), suspected acute pancreatitis, multiple rib fractures with bone marrow emboli, and evidence of both arteriosclerosis and hypertensive nephrosclerosis, in addition to small bowel diverticulosis.

## Discussion

The patient’s underlying ESRD and chronic steroid use likely contributed to significant immune dysfunction, rendering him vulnerable to fulminant bacterial infection [1]. Although his initial presentation was characterized by metabolic derangements associated with ESRD, his clinical status rapidly deteriorated into hypoxemic respiratory failure. Imaging revealed a unilateral consolidation that progressed to bilateral diffuse infiltrates within hours. Despite early broad-spectrum antibiotics, mechanical ventilation, and VA-ECMO, he died within 28 hours of admission.

The multiplex respiratory PCR assay was negative. The clinical features and autopsy findings, including the presence of Gram-positive cocci within necrotic bronchial tissue, suggested MSSA as the primary pathogen. However, false-positive or residual DNA detection by multiplex PCR has been reported, which may mislead initial treatment decisions [10]. This means that PCR tests can sometimes yield positive results due to technical errors or residual DNA from non-active pathogens, potentially leading to inappropriate or unnecessary treatment.

The disease pattern, characterized by hemorrhagic infiltrates, rapid radiological worsening, and bronchial wall destruction, was suggestive of PVL-associated necrotizing pneumonia. Panton-Valentine leukocidin (PVL) is a pore-forming cytotoxin produced by certain strains of *S. aureus*, which causes leukocyte lysis and extensive pulmonary tissue damage [11]. Although classically observed in healthy young individuals following influenza infection, PVL-positive *S. aureus* pneumonia has also been reported in patients with comorbidities and immunosuppression, including ESRD [12]. Notably, severe neutropenia has also been documented in PVL-positive *S. aureus* infections, likely reflecting toxin-mediated leukocyte destruction, further compromising host defense and correlating with poor outcomes [12,13]. In this case, early leukopenia was noted and may have contributed to the fulminant progression. Radiologically, PVL-associated necrotizing pneumonia is characterized by rapid evolution of multilobar consolidations, cavitary lesions, and airway wall destruction on CT scans, often within 24-48 hours [14]. In our case, new bronchial dilatation and peribronchial wall thickening developed within a single day, likely representing necrotic changes secondary to PVL-mediated injury. Clinical recognition of PVL-positive *S. aureus* pneumonia is crucial, given its high mortality, which can exceed 40-50% even with aggressive intensive care [12,14]. Although PVL gene testing was not performed in this case, the clinical and pathological findings do not exclude the possibility of PVL-associated necrotizing pneumonia. Of course, the patient’s profound immunosuppression may have allowed even a non-PVL *S. aureus* strain to cause necrosis, or another staphylococcal toxin may have played a role.

Empirical treatment of severe community-acquired pneumonia (CAP) with necrotizing features should include anti-staphylococcal coverage, including MRSA, until microbiological confirmation is obtained [15]. Agents such as clindamycin or linezolid may provide an additional therapeutic benefit by suppressing PVL toxin production [16]. In this case, the rapid progression of pneumonia outpaced the ability to select and administer broad-spectrum antimicrobial therapy, and the infection proved fatal, underscoring the aggressive nature of this pathogen in immunocompromised hosts.

This case emphasizes the need for heightened clinical vigilance for virulent strains of *S. aureus*, including PVL-producing strains, in rapidly progressive CAP, particularly in vulnerable populations such as those with ESRD or on immunosuppressive therapy. Early recognition, rapid diagnostic workup, initiation of supportive care, and toxin-suppressive therapies are key to improving outcomes in this devastating condition.

## Conclusions

This case highlights the fulminant nature of MSSA pneumonia in an immunocompromised patient with ESRD. Although the initial clinical assessment focused on hyperkalemia and metabolic acidosis, postmortem examination revealed necrotizing pneumonia due to MSSA as the definitive cause of death. The rapid clinical deterioration despite early intervention underscores the importance of recognizing toxin-producing *S. aureus* strains and initiating aggressive therapy without delay. Timely diagnosis and management are critical, yet even with intensive support, outcomes may be poor in such rapidly progressive infections.
